# World Kidney Day 2021 - living well with kidney disease: strengths, weaknesses, opportunities and threats during COVID-19

**DOI:** 10.1590/2175-8239-JBN-2021-0067

**Published:** 2021-05-10

**Authors:** Reginaldo Passoni dos Santos

**Affiliations:** 1 Universidade Estadual do Oeste do Paraná, Hospital Universitário, Departamento de Enfermagem, Cascavel, PR, Brasil.

Dear Editor,

Recently, members of the World Kidney Day (WKD) Steering Committee^1^ described a set of actions to be developed by everyone who is part of the care network for patients with Chronic Kidney Disease (CKD), with the aim of achieving patient-centered wellness. With the theme "Living Well with Kidney Disease", the WKD 2021 focuses on promoting the empowerment of patients with CKD, so they have effective participation in their daily lives.

In parallel, and, despite this, the Sars-CoV-2 infection and mortality rates due to the novel coronavirus disease 2019 (COVID-19) remain persistently high, specially in low-income and lower-middle-income countries. According to the World Health Organization, by March 1, 2021, more than 113 million cases and more than two million deaths by COVID-19 worldwide were recorded^2^.

Given the current pandemic scenario, living well with kidney disease becomes an even greater challenge. COVID-19 increased the factors that promote weaknesses and threats to the wellness of patients with CKD^3^, but there are also strengths and opportunities (Chart 1). Strengths and weaknesses result from situations or aspects that can be controlled by the patients themselves or their care partners. On the other hand, opportunities and threats arise from situations or aspects over which patients do not have full control, as they involve actions carried out by external agents (policy makers, health care institutions and teams, industry partners, and others).

**Chart 1:** SWOT matrix for the factors that may influence the wellness of patients with kidney disease during the COVID-19 pandemic

	**Helpful**	**Harmful**
	to achieve patient-centered wellness	to achieve patient-centered wellness
INTERNAL	**Strengths:**	**Weaknesses:**
	More attention to symptoms;	Loss of clinical follow-up by the health team;
	More attention to self-medication;	Abandonment of treatment;
	Better attention to physicians’ recommendations;	Discouragement for self-care;
	Closer and more confident relationship with the health team;	Non-adherence to health protocols to avoid COVID-19;
	More determinants to the empowerment for self-care;	Negationism of the consequences of COVID-19.
	Greater search for medical evidence-based information.	
**EXTERNAL**	**Opportunities:**	**Threats:**
	Remote contact with the healthcare team through messaging applications;	Greater difficulty in accessing health services;
	Development (by nephrology societies) of specific clinical care guidelines during COVID-19;	Greater need for acute dialysis;
	Development of software for remote clinical monitoring;	More time on the waiting list for kidney transplantation;
	More attention to patients’ mental health;	Mandatory social isolation;
	Improvement of communication and education methods;	Lifestyle changes (work, travel, studies, social activities);
	Improved awareness and knowledge about kidney disease.	Changes in health care priorities by public policy makers;
		Disruption of supply and transport chains (especially drugs and supplies used by patients).

The activities related to WKD 2020 were affected by COVID-19. Although this may occur again in the WKD 2021, the development of all activities planned is encouraged, following the recommendations to avoid contamination by the virus and adapting the actions to the local sanitary conditions.

To improve the care of patients with CKD and acute kidney injury who are affected by COVID-19, the Brazilian Society of Nephrology has published a series of recommendations for good clinical practice, which may guide professionals in the management of cases^4^. In addition, the strategic plan for integrated care of patients with CKD in the post-COVID-19 era must be thought about today. In this sense, the strengths and opportunities created during COVID-19 must be improved and consolidated to ensure the wellness of patients with CKD. Likewise, establishing strategies that can mitigate current weaknesses and threats is essential for patients to continue living well with kidney disease in the post-COVID-19 era.
